# Efficacy of Fecal Microbiota (REBYOTA) in Recurrent Clostridium difficile Infections: A Systematic Review and Meta-Analysis

**DOI:** 10.7759/cureus.58862

**Published:** 2024-04-23

**Authors:** Saivishnu Doosetty, Chukwuemeka Umeh, Wesley Eastwood, Isha Samreen, Ashwin Penchala, Harpreet Kaur, Christine Chilinga, Gagan Kaur, Tamanna Mohta, Sriharsha Nakka, Pavani Tangirala, Sasank Nakka

**Affiliations:** 1 Internal Medicine, KPC Global Medical Center, Hemet, USA; 2 Internal Medicine, Hemet Global Medical Center, Hemet, USA; 3 Internal Medicine, St. George's University, West Indies, GRD; 4 Infectious Disease Control, Hemet Global Medical Center, Hemet, USA

**Keywords:** live-jslm, fecal microbiota, recurrent clostridium difficile, rebyota, rbx2660

## Abstract

Clostridium difficile infections (CDI) are a leading cause of antibiotic-associated diarrhea, and recurrent infections are common despite effective antibiotic treatments. Recurrent CDI causes a significant burden to the patient and healthcare system, which has led to efforts to find an effective treatment to prevent recurrent CDI. Recent studies have shown the efficacy and safety of orally and rectally administered microbiota treatment to prevent recurrent Clostridium difficile. This study systematically reviewed the data on the efficacy and safety of RBX2660 (REBYOTA^®^), the first rectally administered microbiota product to prevent recurrent Clostridium difficile infections approved by the United States Food and Drug Administration (FDA). Our analysis showed that RBX2660 (REBYOTA) effectively prevented recurrent CDI. Patients who received RBX2660 (REBYOTA) were significantly less likely to have recurrent Clostridium difficile than controls eight weeks after treatment. This effect is seen in both those who got one or two doses of RBX2660 (REBYOTA), although the FDA currently approves one dose.

## Introduction and background

Recurrent Clostridium difficile infections (CDI) are defined as the reappearance of symptoms within two to eight weeks after treatment completion and the complete resolution of symptoms [1]. The incidence of recurrent Clostridium difficile is around 25%, and risk factors include age greater than 65 years, IV antibiotic use/hospitalization, gastric acid suppression, lack of antibody-mediated immune response, and severe comorbidities/medical disorders [1]. The pathophysiology of recurrent CDI is presumed to be due to relapse of the initial infecting strain or, less likely, reinfection with a new Clostridium difficile strain [2]. Recurrent CDI causes a significant burden to the patient and healthcare system. A retrospective cohort study at Sherbrooke, Canada, showed that 34% of 1527 patients with recurrent Clostridium difficile infections required readmission to the hospital, 28% developed severe infection, and 4% developed complications [3].

Multiple options exist for treating and preventing recurrent Clostridium difficile infections. Treating Clostridium difficile involves using a course of vancomycin or fidaxomicin [2] while follow-on treatment with rifaximin, bezlotoxumab, and fecal microbiota transplant (FMT) has been shown to decrease Clostridium difficile recurrence [4-8]. In addition, recent studies have shown the efficacy and safety of oral and rectally administered microbiota treatment to prevent recurrent Clostridium difficile infections [9,10]. This study aims to systematically review the data on the efficacy and safety of RBX2660 (REBYOTA^®^), the first rectally administered microbiota product for the prevention of recurrent Clostridium difficile approved by the United States Food and Drug Administration (FDA) in November 2022 [11]. A previous meta-analysis was published using interim results of the PUNCH CD2 study [12]. In addition, the meta-analysis included multiple publications of the PUNCH CD2 study that reported slightly different data as separate studies [12]. With the publication of the final result of the PUNCH CD2 result, which included some corrections to the population definitions and analysis used in the preliminary reports, we updated the meta-analysis to reflect the updated results of the studies.

## Review

Methods

Study Design and Outcome

We designed our meta-analysis according to the Preferred Reporting Items for Systematic Reviews and Meta-Analyses (PRISMA) statement guidelines [13]. We hypothesize that using RBX2660 (REBYOTA) in patients with recurrent Clostridium difficile will decrease recurrence. We did not register our study protocol before the study.

Eligibility Criteria

We included studies that reported Clostridium difficile recurrence and side effects in the intervention and control groups of patients who received RBX2660 (REBYOTA). We included randomized and non-randomized studies, including a study that reported outcomes using a historical control group. Two authors independently reviewed the abstracts after the literature search to assess which papers met our study's inclusion and exclusion criteria.

Literature Search and Data Extraction

Articles were obtained on December 5, 2023, by searching the PubMed and Embase databases with the keywords RBX2660 and recurrent Clostridium difficile, fecal microbiota, live-jslm, and REBYOTA and recurrent Clostridium difficile infection. We did not apply any filters during the search and did not exclude any studies.

Two authors manually and independently reviewed the articles. Information on the number of study participants, the interventions received, and outcomes, including Clostridium difficile recurrence and adverse effects, was extracted onto a datasheet in Microsoft Excel (Microsoft Corporation, Redmond, WA, US 2018).

Methods for Assessing the Quality of Studies and Risk of Bias

We assessed the risk of bias for the randomized controlled trials using the risk-of-bias tool for randomized trials (RoB 2) tool outlined in the Cochrane Handbook [14]. Furthermore, we assessed the risk of bias in the prospective, open-label study with a historical control using the Robins-I tool for assessing the risk of bias in non-randomized intervention studies [15].

Data Synthesis

We performed a meta-analysis by combining the studies' Clostridium difficile recurrence rates and side effects in the intervention and control groups. The open-label clinical trial in our study used a historical control group, which included patients who matched the inclusion and exclusion criteria of the study identified through a retrospective chart review of patients treated with antibiotics for recurrent CDI [16]. Furthermore, the randomized controlled trials in our study reported the modified intention to treat analysis, excluding participants who discontinued treatment before outcome evaluation or had eligibility deviations, as reported in the studies [17,18]. One of the studies reported two independent intervention groups that received one and two doses of RBX2660 (REBYOTA), respectively [18]. We separated the two intervention groups in our primary analysis. We calculated the odds ratio (OR) and 95% confidence interval (CI) for Clostridium difficile recurrence in patients who received RBX2660 (REBYOTA) and controls in each study and the combined composite odds ratio for Clostridium difficile recurrence in the studies. We used the random effect model in our analysis, and the null hypothesis was tested using a Z-test with a p-value of less than 0.05 considered statistically significant. Heterogeneity in study outcomes was tested using the chi-square test and the I2 statistic. A sensitivity analysis was not performed because of the limited number of studies. The analysis was performed using Comprehensive Meta-Analysis Version 3.

Results

Identification of Relevant Studies

**Figure 1:**
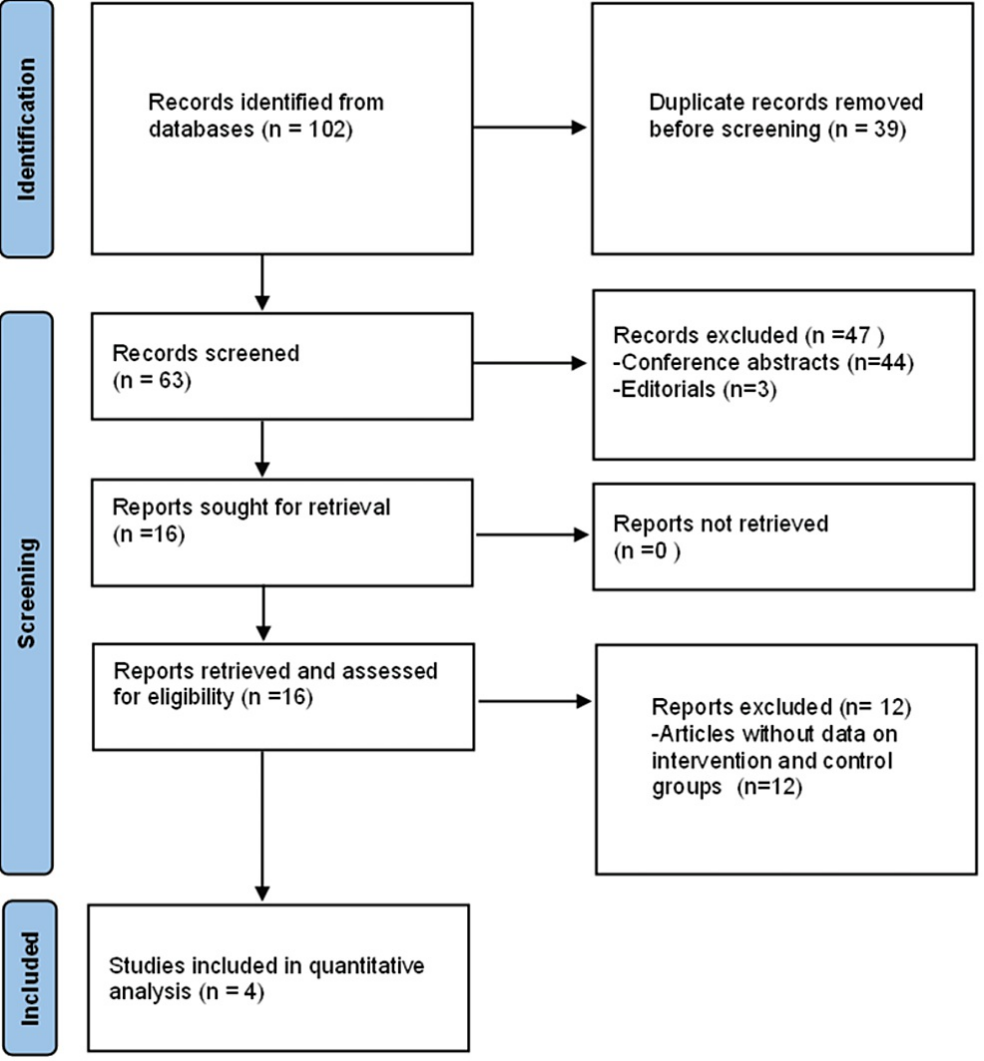
Preferred Reporting Items for Systematic Review and Meta-Analyses guidelines (PRISMA) flowchart of the selection process

Our search produced 102 articles, of which 63 were unique after removing duplicate publications. Two authors independently screened the 63 abstracts to assess if they met our inclusion and exclusion criteria, after which we retrieved and reviewed the full text of 16 papers. Articles excluded include conference abstracts (n=44), editorials (n=3), and articles on RBX2660 but without data on intervention and control groups (n=12) (Figure 1). For articles that reported interim and final results of the same study in different papers, only data from the papers that reported the final results were included. The authors retrieved and had access to the full text of all the relevant studies that met our inclusion criteria (Table 1).

**Table 1:** Studies Included in the meta-analysis

Study	Number of patients	Type of study	Maximum follow-up period	Primary outcome of interest	Result
Orenstein et al., 2022 [16]	217	Open-label trial with historical control group	24 months	Absence of CDI diarrhea without the need for retreatment for 8 weeks after completing study treatment	Patients who received RBX2660 had a 78.9% (112/142) treatment success rate compared to a 30.7% (23/75) for the historical control group
Khanna et al., 2022 [17]	262	Randomized controlled trial	6 months	Absence of CDI diarrhea within 8 weeks of study treatment	The treatment success rate was 70.6% with RBX2660 versus 57.5% with placebo, with an estimated treatment effect of 13.1%
Dubberke et al. 2023a [18]	81	Randomized controlled trial	24 months	Absence of diarrhea and no re-treatment for CDI any time after the first dose until 8 weeks after the second dose of the study treatment	The treatment success rate was 65.8% (25/38) for participants who received one dose of RBX2660, and 44.2% (19/43) for participants who received a placebo in the final modified intention-to-treat (mITT) analysis
Dubberke et al. 2023b [18]	83	Randomized controlled trial	24 months	Absence of diarrhea and no re-treatment for CDI any time after the first dose until 8 weeks after the second dose of the study treatment	The treatment success rate was 62.5% (25/40) for participants who received two doses of RBX2660, and 44.2% (19/43) for participants who received a placebo in the final modified intention-to-treat (mITT) analysis

Risk of Bias Assessment

The randomized controlled trials included in the meta-analysis had a low risk of selection, performance, detection, attrition, and reporting bias. The prospective open-label study included in the meta-analysis was of good quality, with a low risk of selection, performance, attrition, and reporting bias based on the Robins-I tool for assessing the risk of bias in non-randomized intervention studies.

**Figure 2:**
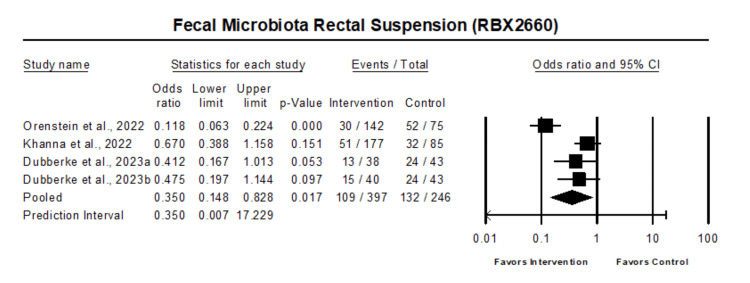
Forest plot of all patients who received fecal microbiota rectal suspension Heterogeneity: Tau2: 0.63; Q-value is 17.2; df: 3 (p=0.001); I-square: 83 Orenstein et al. 2022 [16], Khanna et al. 2022 [17], Dubberke et al. 2023 [18]

The pooled estimate of the effect of fecal microbiota rectal suspension on the recurrence of Clostridium difficile in comparison with the control in 4 studies with 600 patients showed that fecal microbiota rectal suspension was associated with reduced odds of Clostridium difficile recurrence (OR 0.35, 95% CI 0.15 - 0.83, p=0.017) (Figure 2).

**Figure 3:**
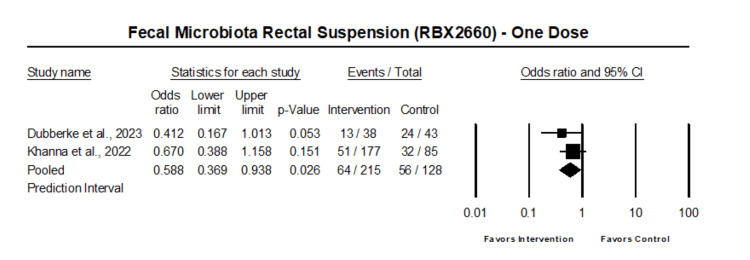
Forest plot of patient who received one dose of fecal microbiota rectal suspension Heterogeneity: Tau2: 0; Q-value is 0.82; df: 1 (p=0.36); I-square: 0 Khanna et al. 2022 [17], Dubberke et al. 2023 [18]

The analysis of those who received one dose of fecal microbiota rectal suspension showed that one dose of fecal microbiota rectal suspension was associated with reduced odds of Clostridium difficile recurrence (OR 0.59, 95% CI 0.37 - 0.94, p=0.026) (Figure 3).

**Figure 4:**
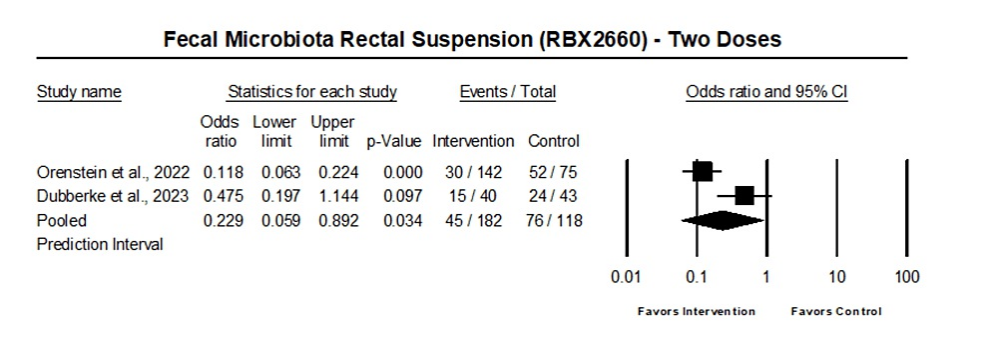
Forest plot of patient who received two doses of fecal microbiota rectal suspension Heterogeneity: Tau2: 0.81; Q-value is 6.3; df: 1 (p=0.012); I-square: 83.1 Orenstein et al. 2022 [16], Dubberke et al. 2023 [18]

The analysis of those who received two doses of fecal microbiota rectal suspension showed that two doses of fecal microbiota rectal suspension were associated with reduced odds of Clostridium difficile recurrence (OR 0.23, 95% CI 0.06 - 0.89, p=0.034) (Figure 4).

**Figure 5:**
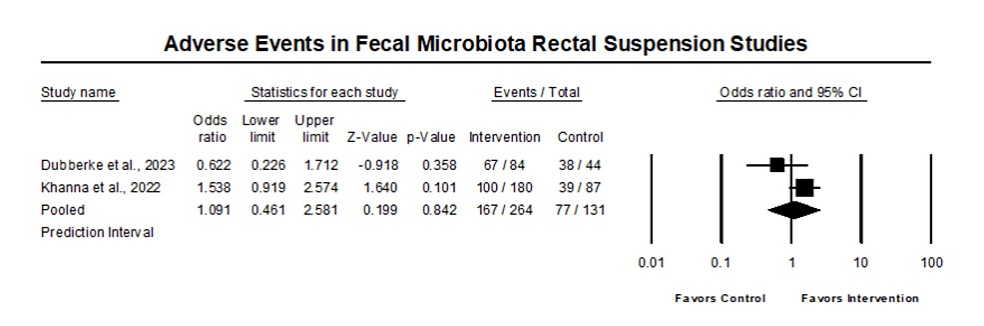
Forest plot of adverse events in fecal microbiota rectal suspension studies Heterogeneity: Tau2: 0.24; Q-value is 2.4; df: 1 (p=0.12); I2: 59.02 Khanna et al. 2022 [17], Dubberke et al. 2023 [18]

In the comparative analysis of all reported side effects, there was no statistically significant difference in side effects in those that received fecal microbiota rectal suspension compared with control (OR 1.09, 95% CI 0.46 - 2.58, p=0.84) (Figure 5).

Discussion

Our analysis showed that RBX2660 (REBYOTA) effectively prevented recurrent CDI. Patients who received RBX2660 (REBYOTA) were significantly less likely to have recurrent Clostridium difficile than controls eight weeks after treatment. This effect is seen in both those who got one dose of RBX2660 (REBYOTA) and those who received two doses. The goal of the fecal microbiota is to restore the gut microbiota to a healthy one by replacing the dysbiotic microbes with healthy microbiota. Thus, the possible reason for decreased Clostridium difficile recurrence is that patients who received RBX2660 had a more rapid and complete gut microbiome recovery within the first seven days after antibiotics treatment than controls [19]. In patients who responded to RBX2660 (REBYOTA), their gut microbiome moved toward becoming similar to the RBX2660 composition within seven days after treatment, with increased similarity noticed up to 60 days after treatment and sustained up to 24 months after treatment [16]. For those who did not respond to RBX2660 treatment, the gut microbiome compositions were similar to those of responders at baseline. However, at 7 and 30 days after treatment, the gut microbiome of the non-responders showed little similarity with the RBX2660 composition. In addition, prior studies showed that patients with recurrent Clostridium difficile successfully treated with RBX2660 at eight weeks had a long-term sustained clinical response with greater than 90% of treatment responders remaining CDI-free at 6, 12, and 24 months [20].

Our study did not show any difference in reported side effects in those who received RBX2660 (REBYOTA) and those who did not. Most adverse events were mild to moderate, primarily related to gastrointestinal disorders, and occurred during the first two weeks after treatment. This finding is consistent with an earlier cumulative analysis of five clinical trials involving 978 participants over 18 years who received one dose of RBX2660 (REBYOTA) after Clostridium difficile recurrence and 87 participants who received a placebo [21]. The study reported adverse effects in 66.4% of those who received RBX2660 (REBYOTA) compared to 60.2% in placebo. Similar to our study, the adverse events were mild to moderate in severity and were predominantly gastrointestinal disorders, including nausea, diarrhea, abdominal pain, and flatulence.

The advantages of RBX2660 (REBYOTA) are generally defined based on the context of decreased recurrence of CDI, cost-effectiveness, and improved quality of life. Various studies have demonstrated the cost-effectiveness and impact on quality of life of RBX2660 (REBYOTA). A study consisting of adult patients with ≥ 1 recurrence after a primary CDI and who had completed ≥ 1 round of antibiotics or had ≥ 2 severe CDI concluded that RBX2660 (REBYOTA) was cost-effective across all sensitivity analyses compared to the standard of care in preventing recurrent CDI [22]. Furthermore, other studies have demonstrated that treatment of recurrent CDI with RBX2660 (REBYOTA) improved patients' quality of life. A secondary analysis of a randomized, double-blinded, placebo-controlled phase 3 study that administered quality of life surveys to participants treated with RBX2660 (REBYOTA) versus a placebo reported significant improvement in the mental domain in those on RBX2660 (REBYOTA) than those receiving a placebo [23].

The challenges associated with RBX2660 (REBYOTA) use include side effects, storage, and cost. The storage of RBX2660 (REBYOTA) and its route of administration could inconvenience both pharmacies and patients. RBX2660 (REBYOTA) cannot be stored in a freezer with other non-fecal microbial transplantation products. It must be administered as a rectal suspension and shipped frozen to pharmacies in a ready-to-administer enema bag. Furthermore, RBX2660 (REBYOTA) costs around $9500 per course in 2023, which may be a barrier, but economic analysis suggested that it is cost-effective compared to the standard of care [22]. RBX2660 (REBYOTA) significantly reduces recurrent CDI, but the cost may be a barrier to access and limit its use in the general population.

Limitations of the study

Our study has certain limitations. First, the small number of studies in the meta-analysis limited the type of analysis that could be done in the study, including sensitivity and sub-group analysis.

Additionally, considerable heterogeneity of the studies in the meta-analysis could have affected our study's conclusion. However, we had similar results in the sub-analysis of those who received one dose of RBX2660 (REBYOTA), where the heterogeneity was zero. Thus, we are confident that our results represent the medication's true effect. Furthermore, we mitigated the impact of heterogeneity in our analysis by using a random-effects model, which provides a more conservative estimate of the intervention effect due to its assumption that the primary studies are heterogeneous.

Implications of results for practice, policy, and future research

RBX2660 (REBYOTA) is safe and effective in reducing recurrent CDI. Though the cost of RBX2660 (REBYOTA) could limit access to the medication, especially for patients with partial or no health insurance coverage, other studies have shown that RBX2660 (REBYOTA) is cost-effective compared to standard care and improves patients' quality of life.

One opportunity for future research is a comparative study of the effectiveness and side effects of RBX2660 (REBYOTA) with other treatment options for recurrent CDI such as fecal microbiota transplant.

## Conclusions

The results of this meta-analysis support the safety and efficacy of RBX2660 (REBYOTA) in preventing recurrent CDI. Our analysis showed that RBX2660 (REBYOTA) effectively prevented recurrent CDI. Patients who received RBX2660 (REBYOTA) were significantly less likely to have recurrent Clostridium difficile than controls eight weeks after treatment. This effect is seen in both those who got one or two doses of RBX2660 (REBYOTA), although the FDA currently approves one dose.
